# Geometry-modulated dipole polarizability of the two-dimensional Mott-Wannier excitons in gate-defined anisotropic quantum dot

**DOI:** 10.1038/s41598-022-19119-2

**Published:** 2022-08-30

**Authors:** A. Poszwa

**Affiliations:** Faculty of Mathematics and Computer Science, University of Warmia and Mazury in Olsztyn, ul. Słoneczna 54, 10-710 Olsztyn, Poland

**Keywords:** Nanoscience and technology, Nanoscale materials

## Abstract

A theoretical investigation on neutral excitons confined to a mono-layer (ML) semiconductor transition metal dichalcogenide (TMDC) materials under the influence of elliptically deformed gate induced confining potential is presented. It has been shown that the anisotropy of the confinement induces the anisotropy of linear response of the system on in-plane external electric field. The linear response is expressed in terms of principal moments of the static dipole polarizability tensor. In this manner the direction-dependent polarizability of the system can be fully controlled by tuning the parameters of gate-induced confining potential. The components of the polarizability tensor are determined using finite-field method based on the exact diagonalization of the electron-hole Hamiltonian including confining potential, Coulomb electron-hole interaction and an external electric field, within effective mass approximation, close to the K-points of the first Brillouin zone of a single-layer MX$_2$ material. The useful scaling relations for energies and dipole polarizabilities as functions of material parameters have been found. The influence of the anisotropy of the confining potential on correlated behavior of charge distribution inside the neutral system has also been demonstrated.

## Introduction

The correlation between geometry and electronic properties in two-dimensional semiconductor structures has recently become an active area of theoretical and experimental research due to its potential application for electronic purposes. The interplying between electronic properties and geometry appears as an efficient tool for manipulation of band structure or transport properties what allows for the design of electronic nano-devices with a desired functionality. In graphene systems, the coupling between geometry and the electronic band structure is the most visible for graphene nanoribbons (GNRs) and carbon nanotubes (CNTs)^1,2^. In the case of GNRs, the electronic states strongly depend on the width and on the edge structure of the ribbon. In the case of CNTs the electrical properties essentially depend on the diameter and on the shape of the edges. One of the most recognized examples of ballistic transport devices exhibiting correlation between geometry and electronic properties is the geometric diode. In the geomeric diode the electric current rectification is obtained due to geometrical asymmetry^3,4^. In many materials, energy bands posses a discrete number of inequivalent local minima or maxima for specific values of momenta. The minima usually known as *valleys* seem to be promising candidates for components of pseudospin or a binary variable^5,6^. The separation of charge current composed of electron states belonging to only one valley can be regarded to as valley polarization. For graphene in particular, several schemes have been proposed to achieve valley-current filtering depending on geometrical deformation. At the field of geometry-induced effects also strain-induced effects in graphene has been the topic of a large number of theoretical works aimed at understanding the impact of controlled geometrical deformations on electronic properties^7–13^.

Recent advances in the epitaxial growth of 2D crystals have opened up new opportunities towards novel devices based on van der Waals heterostructures in which TMDCs play a major role. Two-dimensional TMDC materials, such as MoS$_2$, WS$_2$, MoSe$_2$, and WSe$_2$, have received extensive attention in the past decade due to their extraordinary electronic, optical and thermal properties^14–16^. They are considered as ideal materials for next-generation electronics, photonic and opto-electronic devices, relying on ultimate atomic thicknesses^17,18^. The bandgap of semiconducting TMDCs can be sized by varying the number of layers, and it can be changed from indirect to direct approaching the single layer. This tunable bandgap in TMDCs is accompanied by a strong photoluminescence and large exciton binding energy, making them promising candidate for a variety of opto-electronic devices, including solar cells, photo-detectors, light-emitting diodes, and photo-transistors^19–22^. Similarly to traditional semiconductor heterostructures quantum dots (QDs) can be formed on atomically thin ML TMDCs materials by applying a gate voltage to tune the local band structure^23^. Gate-defined QDs, in addition provide an efficient tool to tune electrically the confinement geometry and the strength. The realization of a quantum dot device has been obtained from the nanosheet on a Si/SiO$_2$ substrate, and quantum dot confinement has been achieved by the top gate technique^24^. The fabrication of single quantum dots defined by gates has been reported recently for bilayer and monolayer WSe$_2$ and MoS$_2$ materials and discrete levels at temperatures up to 10 K have been observed^25,26^. The Coulomb blockade in single and coupled dots in high quality single layer MoS$_2$ and the Shubnikov-de Haas oscillations occurring at magnetic fields as low as 3.3T have been observed^24^. Intrinsic exciton-state mixing and nonlinear optical properties, particulary the mechanism of second harmonic generation in TMDCs monolayers have been recently investigated on the basis of symmetry analysis^27^. Many other investigations based on several approaches including tight binding method, ab initio calculations, a many-body Bethe-Salpeter equation, $\varvec{k}\cdot \varvec{p}$ perturbative method and effective mass approximation have been performed on properties of excitons formed in 2D semiconductor materials, also in the presence of confinement^28–35^. One should be also added that the Coulomb-exchange interaction, having an important influence on the energy structure of the 2D excitons, can be separated into the long-range and the short-range parts. In particular, the long-range exchange interaction has quantum electrodynamic nature, by the analogy to the exchange interaction in a positronium^36^. A theoretical study on the long-range exchange interaction in excitons has been performed treating the bright exciton as a microscopic dipole which produces an electric field and the backaction of this field on the exciton leads to the long-range electron-hole exchange interaction. Formally this treatment corresponds to the decomposition of the Coulomb interaction up to the dipole term and calculation of matrix element of the dipole term using the antisymmetrized Bloch functions^28^.

The purpose of this paper is a theoretical investigation of anisotropic quantum confinement effect on the linear response on external electric field of a neutral Mott-Wannier exciton formed inside the gate-defined quantum dot, for different geometries of the dot. The dot shape can be controlled electrostatically by appropriate system of metallic electrodes^25,26^. The electrodes generate the confining potential trapping additional electron added into conduction band (CB) as well as the hole in the valence band (VB). We model the confinement using non-centrosymmetric parabolic potential with the deformation of an elliptic type. In particular, we study the effect of the dot geometry on quantum properties of the Mott-Wannier exciton in terms of static dipole polarizability tensor and correlated probability distribution function of the electron-hole pair confined to the dot. Although obtained results can be directly linked with any ML TMDC system, due to relevant scalling relations derived in this paper, we explicitly demonstrate the dependence of static dipole polarizabilities on the dot geometry using material parameters appropriate for MoS$_2$ ML structure. One should be noted that the static dipole polarizability is the property of atomic or molecular system that determines the behavior of neutral particles in the interaction with other particles such as in collision phenomena. The polarizability, in particular, allows for effective description of a long-range van der Waals exciton-exciton interaction or exciton-electron scattering in terms of the dipol-dipol and dipol-monopol interaction, respectively^32^. Moreover, the dipole polarizability is related to the dielectric constant and the reflection coefficient. Thus, in order to determine these parameters theoretically, the excitonic contribution to the total dielectric polarization should also be taken into account^37^.

### Model

**Figure 1:**
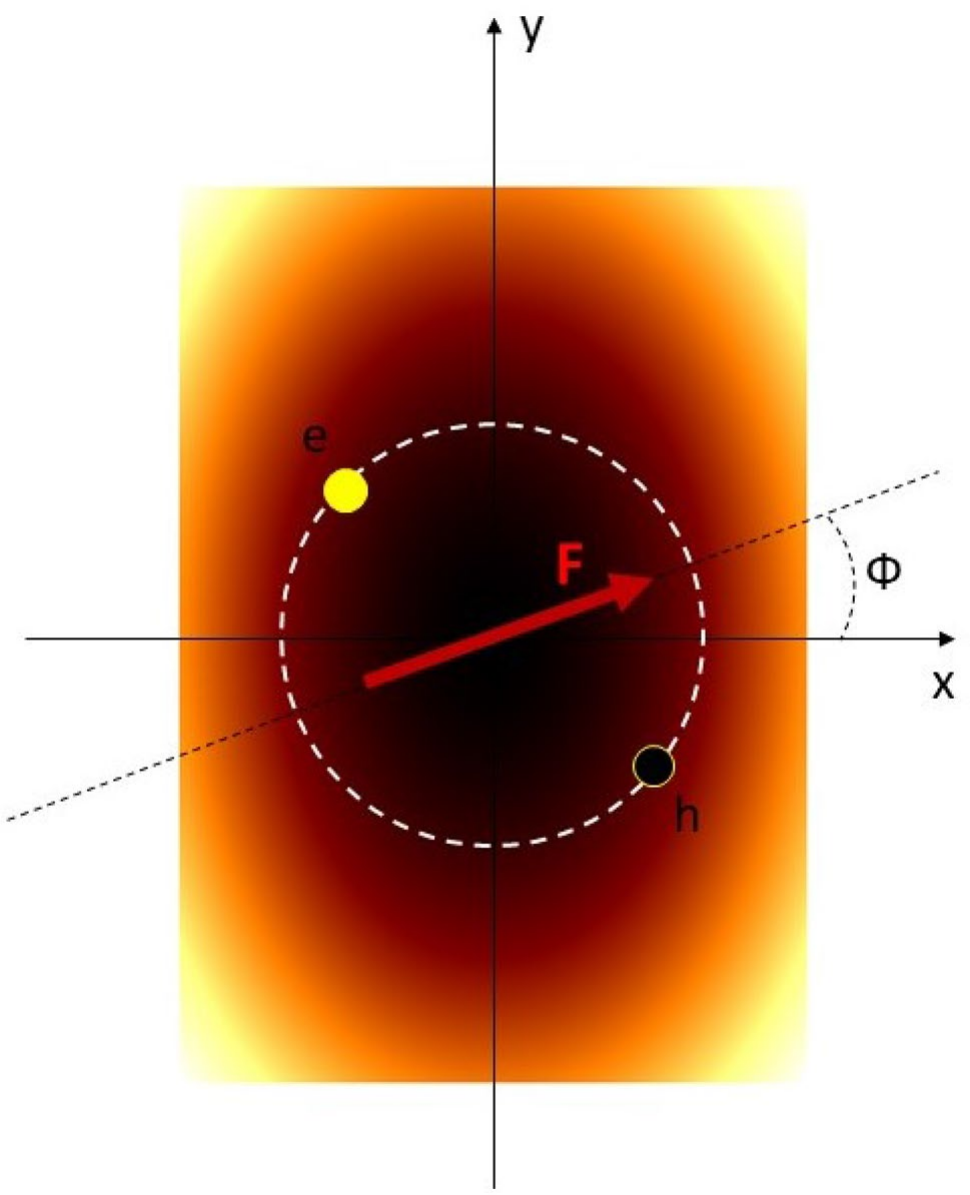
Sketch of the system under study. The electron-hole pair is confined to the 2D elliptically deformed parabolic potential. Bright (dark) region correspond to high (low) values of the potential. The red arrow indicates external in-plane electric field, pointing in direction given by angle $\Phi$.

**Figure 2:**
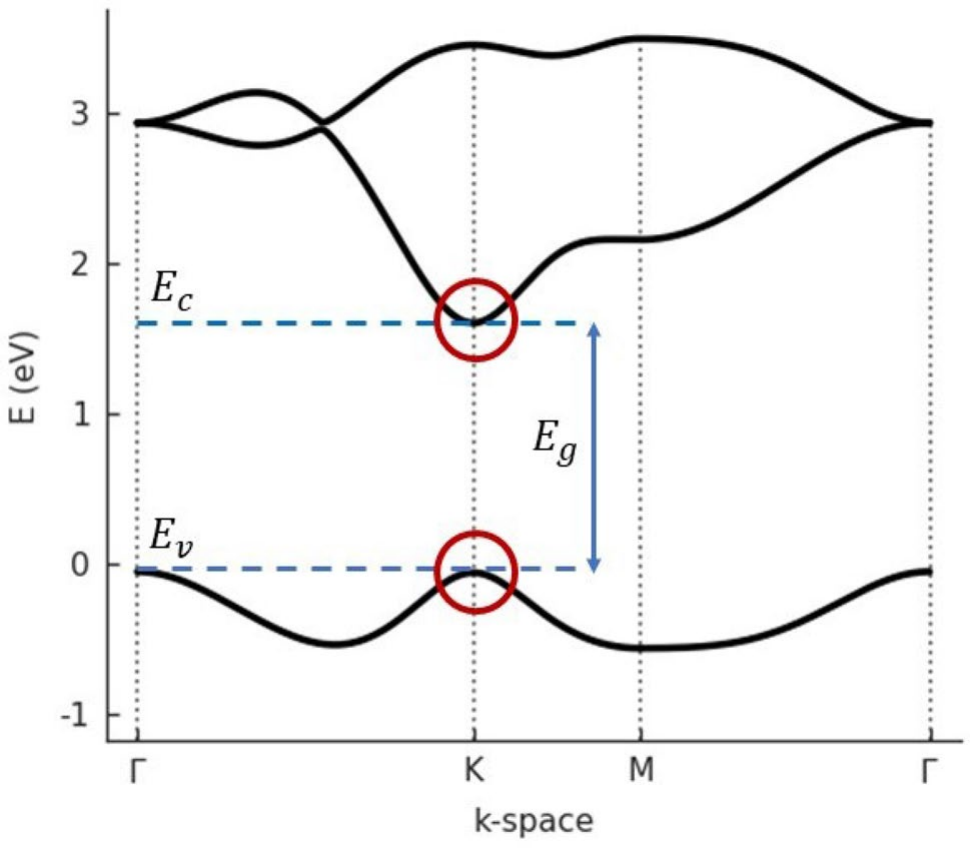
Band structure of monolayer MoS$_2$ calculated within three-band tight-binding model^43^. Conduction (valence) band edge is given by $E_c (E_v)$. The free particle band gap $E_g=1.6$eV. The effective masses at K-point are $m_e=0.60, m_h=0.54$ in units of free electron mass. The reduced mass $\mu =0.28$. Red circles denote regions where the effective mass approximation is used. The band structure has been calculated with the help of Pybinding Python package^44^.

The system under study is sketched in Fig. 1. Due to anisotropy of confining potential the circular symmetry of the system, supposed within the effective mass approximation, is broken and in a consequence the angular momentum is not conserved quantity. The anisotropy leads to the mixing of states with different angular momentum quantum numbers. However, in the absence of external electric field the parity quantum number is still a good quantum number and only the states with even or odd angular momentum quantum numbers are mixed. If the external electric field is applied then the inversion symmetry is also broken and all the angular momentum eigenstates are mixed. For this reason the use of a more sophisticate method such as tight binding atomistic approach or Bethe-Salpeter equation based treatment including the noncentrosymmetric confinement is highly limited. We can see in Fig. 2 that parabolic-like behavior of bands is strongly pronounced close to the K-point. This is true for every ML TMDCs and the effective mass approximation used in this work based on this observation is optimal.

The confining potential acting on a particle with the mass *m* is supposed in the form of anisotropic parabolic potential,

1 $$\begin{aligned} \mathsf V_{\text {conf}}(\varvec{r})=m\mathsf U_{\text {conf}}(\varvec{r}), \end{aligned}$$

where $\varvec{r}=\big [x,y\big ]$ is the coordinates vector of the electron and

2 $$\begin{aligned} \mathsf U_{\text {conf}}(\varvec{r})=\frac{1}{2}\left( \omega _x^{2}x^{2}+\omega _y^{2}y^{2}\right) , \end{aligned}$$

where oscillator frequencies $\omega _x$ and $\omega _y$ are different, in a general case. The dynamics of the electron in the CB and VB in the presence of confining potential, given by Eq. (1) and in the presence of in-plane external electric field, $\varvec{F}=F\big [\cos \Phi ,\sin \Phi \big ]$, oriented along the direction given by the angle $\Phi$, is described by the Hamiltonian $\mathsf H_{\text {e}}^{\text {c}}$ and $\mathsf H_{\text {e}}^{\text {v}}$, respectively. The Hamiltonians in the effective mass approximation are defined as

3 $$\begin{aligned} \mathsf H_{\text {e}}^{\text {c}}-E_c= & {} -\frac{\hbar ^{2}}{2m_{e}^{c}}\nabla _{ec}^{2} +m_{e}^{c}\mathsf U_{\text {conf}}(\varvec{r}_{ec})-q_e\varvec{F}\cdot \varvec{r}_{ec}, \end{aligned}$$

4 $$\begin{aligned} \mathsf H_{\text {e}}^{\text {v}}-E_v= & {} -\frac{\hbar ^{2}}{2m_{e}^{v}}\nabla _{ev}^{2} +m_{e}^{v}\mathsf U_{\text {conf}}(\varvec{r}_{ev})-q_e\varvec{F}\cdot \varvec{r}_{ev}, \end{aligned}$$

where $E_{c(v)}$ is the edge energy of CB (VB), $q_e$ is the electron charge $(q_e<0)$, $m_{e}^{c(v)}$ is the electron effective mass in the CB (VB), where $m_e^{c}>0$ $(m_e^{v}<0)$. In accordance with the commonly known interpretation, the absence of the electron in filled VB may be treated as a quasi-particle (hole) in the VB with the effective mass $m_h\equiv -m_e^{v}>0$ and the charge $q_h=-q_e>0$. Introducing the notation $e=\mid q_e\mid$, $m_e^{c}\equiv m_e, r_{ec}\equiv r_e, r_{ev}\equiv r_h$, we can rewrite above equations in the forms

5 $$\begin{aligned} \mathsf H_{\text {e}}^{\text {c}}-E_c=\mathsf h_{\text {e}}, \mathsf H_{\text {e}}^{\text {v}}-E_v=-\mathsf h_{\text {h}}, \end{aligned}$$

where the electron and the hole hamiltonians read

6 $$\begin{aligned} \mathsf h_{\text {e}}= & {} -\frac{\hbar ^{2}}{2m_{e}}\nabla _{e}^{2} +m_e\mathsf U_{\text {conf}}(\varvec{r}_{e})+e\varvec{F}\cdot \varvec{r}_{e}, \end{aligned}$$

7 $$\begin{aligned} \mathsf h_{\text {h}}= & {} -\frac{\hbar ^{2}}{2m_{h}}\nabla _{h}^{2} +m_h\mathsf U_{\text {conf}}(\varvec{r}_{h})-e\varvec{F}\cdot \varvec{r}_{h}. \end{aligned}$$

At this point we can note that observed experimentally excitonic spectra are a result of excitation of the electron from VB to CB, by photons. Thus, the excitonic energy levels correspond to the energy difference between the states in VB from which the electron is excited and the states in CB to which the electron is excited. This means that the spectrum, in the first approximation, is related to the eigenvalues of the operator $\mathsf H_{\text {e}}^{\text {c}} -\mathsf H_{\text {e}}^{\text {v}}$. Taking into account electrostatic Coulomb interaction between the electron and the hole,

8 $$\begin{aligned} \mathsf V_{\text {eh}}=-\frac{e^{2}}{\varepsilon \mid \varvec{r}_e-\varvec{r}_h \mid }, \end{aligned}$$

where $\varepsilon$ is the electric permittivity, we can define the excitonic Hamiltonian within the effective mass approximation as

9 $$\begin{aligned} \mathsf H_{\text {exc}}=\mathsf H_{\text {e}}^{\text {c}} -\mathsf H_{\text {e}}^{\text {v}}+\mathsf V_{\text {eh}}=E_c-E_v +\mathsf h_{\text {e}}+\mathsf h_{\text {h}}+\mathsf V_{\text {eh}}. \end{aligned}$$

We note that the above Hamiltonian is independent of the position of the *zero-point* energy in the energy scale, relatively to which the energy bands edges $E_c, E_v$ are defined. Finally, the two-particle Hamiltonian describing the exciton as a pair of two interacting particles (electron-hole), in a more explicit form reads

10 $$\begin{aligned} \mathsf H_{\text {exc}}(\varvec{r}_e,\varvec{r}_h)=E_g-\frac{\hbar ^{2}}{2m_{e}}\nabla _{e}^{2} -\frac{\hbar ^{2}}{2m_{h}}\nabla _{h}^{2}+\mathsf V_{\text {conf}}(\varvec{r}_{e}) +\mathsf V_{\text {conf}}(\varvec{r}_{h})+e\varvec{F}\cdot (\varvec{r}_{e} -\varvec{r}_{h})+\mathsf V_{\text {eh}}, \end{aligned}$$

where $E_g=E_c-E_v$ is the free-particle bandgap. Note that the free-particle bandgap becomes the optical bandgap, when the exciton is formed. The optical bangap is the energy distance from $E_v$ to the lowest excitonic bound state (1*s*), due to the Coulomb interaction between the electron and a hole. The eigenvalue problem,

11 $$\begin{aligned} \mathsf H_{\text {exc}}(\varvec{r}_e,\varvec{r}_h)\Psi _{\text {exc}}(\varvec{r}_e,\varvec{r}_h)= E_{\text {exc}}\Psi _{\text {exc}}(\varvec{r}_e,\varvec{r}_h) \end{aligned}$$

can be effectively solved after introducing the center-of-mass vector $\varvec{R}=(m_e\varvec{r}_e+m_h\varvec{r}_h)/(m_e+m_h)$ and the relative motion vector $\varvec{r}=\varvec{r}_e-\varvec{r}_h$, that separates the two-particle Hamiltonian (10) into the sum of two independent parts $\mathsf H_{\text {exc}}(\varvec{R},\varvec{r})=\mathsf H_{\text {c.m.}}(\varvec{R})+ \mathsf H_{\text {rel}}(\varvec{r})$. In a consequence $E_{\text {exc}}=E_{\text {c.m.}}+E_{\text {rel}}$ and $\Psi _{\text {exc}}(\varvec{r}_e,\varvec{r}_h)=\Psi _{\text {c.m.}}(\varvec{R})\Psi (\varvec{r})$. Details of calculations are given in the Appendix. Since the external electric field does not affect the c.m. motion (due to opposite signs of the charges of two constituents), we consider only the relative motion part of the total Hamiltonian. It is convenient for further analysis to introduce atomic units: $a_0=\frac{\hbar ^{2}}{m_0e^{2}}\simeq 0.529\text{\AA}$ as unit of length, $E_0=\frac{m_0e^{4}}{\hbar ^{2}}\simeq 27.2$eV as unit of energy and $F_0=\frac{m_0^{2} e^{5}}{\hbar ^{4}}\simeq 5.14\times 10^{11}$V/m as unit of electric field. Finally the relative motion Schrödinger equation, in polar coordinates ($r,\vartheta$) introduced in the plane of the system, takes the form:

12 $$\begin{aligned} \Big [-\frac{1}{2\mu }\nabla _r^{2}-\frac{1}{\varepsilon r}+\mu \Omega ^{2}r^{2}\Big (\frac{a^{2}}{1+a^{2}} +\frac{1-a^{2}}{1+a^{2}}\sin ^{2}\vartheta \Big )+\eta r(\cos \Phi \cos \vartheta +\sin \Phi \sin \vartheta )\Big ]\Psi =E\Psi , \end{aligned}$$

where we have introduced following dimensionless parameters: $\mu =m_r/m_0$, where $m_r=m_em_h/(m_e+m_h)$ is the reduced mass of the system, $\Omega =\hbar \omega /E_0$, where $\omega ^{2}=(\omega _x^{2}+\omega _y^{2})/2$ is average square of the oscillator frequency, $a=\omega _x/\omega _y$ is the anisotropy parameter, $\eta =F/F_0$ and $E=E_{\text {rel}}/E_0$. Here $r=\mid \varvec{r}_e-\varvec{r}_h\mid /a_0$. The energy eigenvalues are functions of the system parameters. Multiplying Eq. (12) by $\mu$ and using appropriate scaling of radial variable $(r\varepsilon /\mu \rightarrow r)$, we can find usefull scaling relation for energies

13 $$\begin{aligned} E(\Omega ,\eta ,\mu ,\varepsilon )=\frac{\mu }{\varepsilon ^{2}} E\Bigg (\frac{\Omega \varepsilon ^{2}}{\mu },\frac{\eta \varepsilon ^{3}}{\mu ^{2}},1,1\Bigg ), \end{aligned}$$

that in the case ($\Omega =0, \eta =0$) reduces to the well known formula for energies of an ideal 2D hydrogen atom,

14 $$\begin{aligned} E_n(0,0,\mu ,\varepsilon )=-\frac{\mu }{\varepsilon ^{2}}\frac{1}{2N_n^{2}},N_n=n-\frac{1}{2}, \end{aligned}$$

where $n=1,2,\ldots$ and energies are given in the units of $E_0$. By expanding both sides of the scaling relation, given by Eq. (13) in powers of $\eta ^{2}$ and equating coefficients with equal powers of $\eta ^{2}$ we obtain, in particular, a scaling relation for the dipole polarizability (see following section)

15 $$\begin{aligned} \alpha (\Omega ,\mu ,\varepsilon )=\mu ^{-3}\varepsilon ^{4}\alpha \Big (\frac{\Omega \varepsilon ^{2}}{\mu },1,1\Big ). \end{aligned}$$

In the case of unconfined system ($\Omega =0$) we obtain $\alpha =\mu ^{-3}\varepsilon ^{4}\alpha ^{2D}$, where $\alpha ^{2D}=21/128$ is the polarizability of an ideal 2D H-like atom^38^. The scaling (15) gives in particular a direct relation between Mott-Wannier exciton polarizabilities for two arbitrary ML MX$_2$ materials, $\alpha _1/\alpha _2=(\mu _2/\mu _1)^{3}(\varepsilon _1/\varepsilon _2)^{4}$.

#### The finite-field method

In order to determine components of the dipole polarizability tensor we apply the finite-field method described below. The expectation value of the dipole moment of the system in the presence of external electric field is the sum of the permanent dipole moment and the contribution induced by the field $\varvec{F}$,

16 $$\begin{aligned} P_i(\varvec{F})=P^{(0)}_i+\alpha _{ij}F_j+\frac{1}{2}\gamma _{ijk}F_jF_k+\cdots , \end{aligned}$$

where $\alpha _{ij}$ is the polarizability tensor, $\gamma _{ijk}$ is the first hyperpolarizability and the summation over *j*, *k* is supposed. On the other hand according to the Hellman-Feynman theorem we can write^39^

17 $$\begin{aligned} P_i(\varvec{F})=-\frac{\partial E}{\partial F_i}=-\Big (\frac{\partial E}{\partial F_i}\Big )_0-\Big (\frac{\partial ^{2} E}{\partial F_i\partial F_j}\Big )_0F_j-\frac{1}{2}\Big (\frac{\partial ^{3} E}{\partial F_i\partial F_j\partial F_k}\Big )_0F_jF_k-\cdots , \end{aligned}$$

where $E(\varvec{F})$ is the energy of the system as function of electric field. Since the c.m. motion is not affected by the constant uniform electric field, *E* denotes effectively the relative motion energy. By comparing Eqs. (16) and (17) one obtains

18 $$\begin{aligned} \alpha _{ij}=-\Big (\frac{\partial ^{2} E}{\partial F_i\partial F_j}\Big )_0. \end{aligned}$$

The second-order derivatives of the energy with respect to the electric field can be defined by the Taylor expansion of the field-dependent energy $E(\varvec{F})$,

19 $$\begin{aligned} E(\varvec{F})=E(0)+\frac{1}{2}\Big (\frac{\partial ^{2} E}{\partial F_i\partial F_j}\Big )_0F_iF_j+\cdots , \end{aligned}$$

that contains only even powers of the field magnitude, due to the parity conservation in the field-free system. The last expansion in the low-field limit is equivalent to the perturbation expansion and the components of the dipole polarizability tensor are conventionally obtained using the perturbative approach. For an ideal 2D hydrogen atom the perturbative expansion is known up to third-order in $F^{2}$^40^,

20 $$\begin{aligned} E^{2D}(F)=-2-\frac{21}{2^{8}}F^{2}-\frac{22947}{2^{20}}F^{4}-\frac{48653931}{2^{31}}F^{6}-\cdots \end{aligned}$$

and the scalar (due to circular symmetry) polarizability of 2D H-like atom is $\alpha ^{2D}=21/128$^38^. However, in the problem considered in this paper the use of the perturbation method is very inefficient since we do not have analytical eigen-solutions of the unperturbed problem, summation over which must be performed in the second-order perturbation theory. Instead, we perform exact diagonalization of the total Hamiltonian including an electric field for several different values of weak electric field $(F_{(i)}, i=1,2,\ldots )$. This gives corresponding energies ($E_{(i)}$, i=1,2,...) In the next step we construct the system of linear equations

21 $$\begin{aligned} A_{0}+A_{1}F_{(i)}^{2}+A_{2}F_{(i)}^{4}+A_{3}F_{(i)}^{6}+\cdots =E_{(i)},\quad i=1,2,\ldots \end{aligned}$$

for unknown coefficients $A_{i}$ and with given r.h.s. At this point we note that axes *x*, *y* are chosen according to the symmetry of the system. In a consequence the polarizability tensor is diagonal with respect to these axes. Thus, it is sufficient to calculate principal moments of the polarization tensor taking the electric field vector oriented along the axis *x* and *y*, separately. Taking the vector of electric field as $\varvec{F}=(F,0)$ and solving the linear system (21) we equate the coefficents of the perturbation expansion given by Eq. (19) with the coefficients $A_{i}$: $E(0)=A_0, \alpha _{xx}=-2A_1,\ldots$ Similarly, taking $\varvec{F}=(0,F)$ we obtain the component $\alpha _{yy}$. In this manner we can reconstruct the perturbation series without summation over states. In particular, as the test of the method, the perturbative expansion corresponding to the 2D hydrogen problem, given in Eq. (20) has been reconstructed with high precision. Finally, the magnitude of the dipole polarizability for any direction is given by

22 $$\begin{aligned} \alpha (\Phi )=\alpha _{xx}\cos ^{2}\Phi +\alpha _{yy}\sin ^{2}\Phi , \end{aligned}$$

where the angel $\Phi$ indicates the spatial direction. We note that the finite-field method described above has been succesfully employed for determination of the relativistic magnetic susceptibilities of 3D Dirac H-like atoms^41^.

## Results and discussion

**Figure 3:**
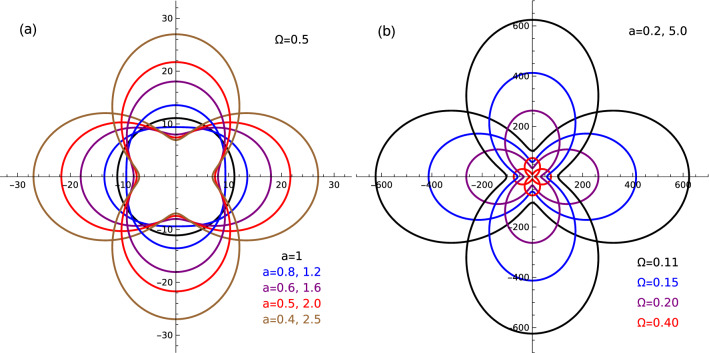
Ground state static dipole polarizabilities of Mott-Wannier excitons for different levels of anisotropy of confinement: (**a**) dependence on anisotropy parameter $a=\omega _x/\omega _y$ with fixed confining strength $\Omega =0.5$; left (right) column listing anisotropy parameters *a* corresponds to the deformation along *x* (y) axis; (**b**) dependence on confining strength $\Omega$ with fixed anisotropy parameter $a=\omega _x/\omega _y=0.2$ (deformation along *x* axis) and $a=\omega _x/\omega _y=5$ (deformation along *y* axis). Material parameters of MoS$_2$ monolayer has been used: $\mu =0.28$ in units of free electron mass and $\varepsilon =5$. In the case of unconfined system ($\Omega =0$) polarizability is given by $\alpha (\Omega =0,\mu ,\varepsilon )=\mu ^{-3}\varepsilon ^{4}\alpha ^{2D}$, where $\alpha ^{2D}=21/128$ is the polarizability of ideal 2D hydrogen atom. The value of polarizability for any direction is equal to the distance of the point on the curve from origin. Polarizabilities are given in units of $a_0^{3}$, where $a_0\simeq 0.529\text{\AA}$ is the Bohr radius.

Figure 3a presents dependence of static dipole polarizability of the 2D Mott-Wannier exciton in the ground state, formed in MoS$_2$ ML, for several values of the deformation parameter $a=\omega _x/\omega _y$, describing a degree of elliptical deformation of the confinement. The strength of the confinement is given by the dimensionless parameter $\Omega$, that is fixed on the value 0.5. We can see that in the case of circular symmetry of the system ($a=1$) the polarizability becomes a scalar and the symmetry of the system is reflected in the polarizability dependence on the angle $\Phi$, that is isotropic in this case. For $a<1$, corresponding to the deformation extended along the *x* direction the polarizability becomes higher in the *x* direction that can be understood since the lower confinement in this direction causes the electron-hole distance to be larger relatively to the distance along the *y* direction and the response on the external electric field becomes higher in the *x* direction, that is reflected in higher polarizability for $\Phi \approx 0$. The situation is reversed in the case of $a>1$, when $\alpha$ becomes maximal for $\Phi \approx \pi /2$. Figure 3b presents the direction-dependence of static dipole polarizability of 2D Mott-Wannier exciton for several values of the confinement strength $\Omega$, for fixed values of the anisotropy parameter $a=0.2$ (quantum dot extended in *x* direction) and $a=5$ (quantum dot extended in *y* direction, with the same degree). We can see that in this case the polarizability is simply rotated by the angle $\pi /2$ relatively to the case of the *x*-extended quantum dot, that is a consequence of the symmetry transformation of the confining potential connecting the cases ’*a*’ and ’1/*a*’. One can also see that the polarizability increases with decreasing confinement strength.

**Figure 4:**
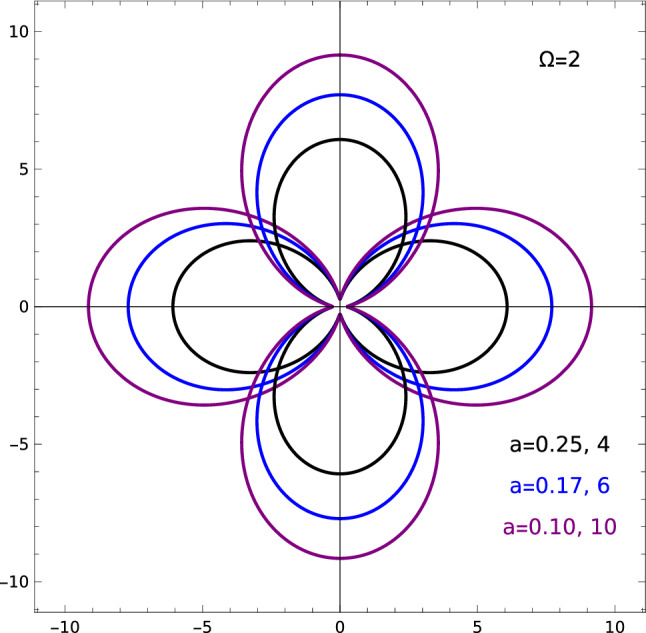
Static dipole polarizabilities of Mott-Wannier exciton in first excited state for different levels of anisotropy of confinement; left (right) column listing anisotropy parameters *a* corresponds to the deformation along *x* (y) axis. Material parameters of MoS$_2$ monolayer has been used: $\mu =0.28$ in units of free electron mass and $\varepsilon =5$. The value of polarizability for any direction is equal to the distance of the point on the curve from origin. Polarizabilities are given in units of $a_0^{3}$, where $a_0\simeq 0.529\text{\AA}$ is the Bohr radius.

Figure 4 presents dependence of static dipole polarizability of the 2D Mott-Wannier exciton in the first excited state, formed in MoS$_2$ ML, for several values of the deformation parameter $a=\omega _x/\omega _y$ and for fixed confinement strength $\Omega =2$. One can note that the dipole polarizability of any H-like system in excited state is greater than in the ground state since the electron is weaker bounded when it is in an excited state and as a consequence the system is more susceptible on the influence of an external electric field. The same concerns the dependence on the atomic number $Z_A$. The greater $Z_A$, the greater Coulomb attraction inside the system and the lower polarizability. The exact dependence is $\alpha \sim Z_A^{-4}$, for both 2D and 3D H-like atoms^38^. We can note that, similarly to the effect of increasing atomic number $Z_A$ in H-like atom, a large confinement ($\Omega =2$) strongly reduces the polarizability of the exciton.

**Figure 5:**
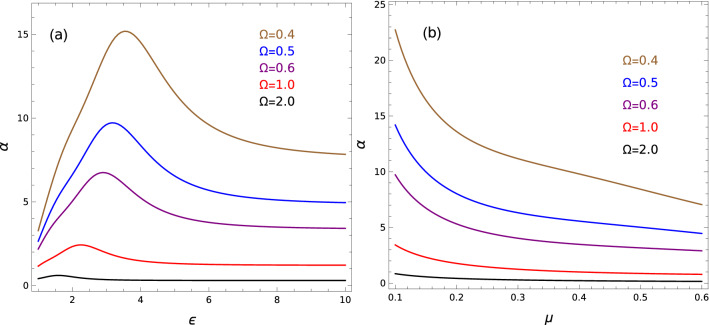
Ground state static dipole polarizabilities (in units of $a_0^{3}$) of Mott-Wannier exciton for isotropic confinement ($a=1$) for several values of confinement strength ($\Omega$): (**a**) dependence on electric permittivity with fixed reduced mass of the system $\mu =0.28$; (**b**) dependence on reduced mass with fixed electric permittivity $\varepsilon =5$.

It is worth to note that contrary to the electron confined in the atom due to Coulomb interaction with a nucleus, the gate-defined confinement opens up the possibility of controlling the polarizability of the excitonic system by changing confinement strength and its geometry. More precisely, one can be observed the existence of a competition between the effective Coulomb interaction between electron and hole and the geometric confinement in producing polarizability. We note that pure Coulomb attraction between electron and hole is effectively modified by material parameters $\mu$ and $\varepsilon$ (see Appendix). This coupling leads to the two-parameter dependence of the excitonic energies and dipole polarizabilities, as is given in Eqs. (13) and (15). On the other hand the parameters $\mu$ and $\varepsilon$ modify also the strength of the confinement, leading to effective strength $\Omega _{\text {eff}}=\Omega \varepsilon ^{2}/\mu$. Figure 5a presents dependence of dipole polarizability $\alpha$ on parameter $\varepsilon$ with fixed parameter $\mu =0.28$, for several confinements in isotropic case ($a=1$). One can see that the reduction of the Coulomb attraction via increasing $\varepsilon$ leads to increasing $\alpha$, up to some $\varepsilon$, at which $\alpha$ becomes maximal. A further increase in $\varepsilon$ causes a decrease in polarizability, what can be understood based on scaling relation (15). We can see that in general $\alpha \sim \varepsilon ^{4}$, what is responsible for increasing $\alpha$ with growing $\varepsilon$, while effective confinement strength $\Omega _{\text {eff}}\sim \varepsilon ^{2}$, what in turn is responsible for decrease of $\alpha$ with increasing $\varepsilon$, due to growing confinement. This competition between Coulomb interaction and confinement possesses some *critical* point, corresponding to the maximum of $\alpha$. Figure 5b presents dependence of $\alpha$ on the parameter $\mu$, with fixed parameter $\varepsilon =5$. We can see that $\alpha$ discloses a monotonic decrease as a function of $\mu$. This behavior may be also linked to the scaling relation (15). In this case, the decrease of $\alpha$ with growing $\mu$, due to the factor $\mu ^{-3}$, exceeds the increase of $\alpha$ caused by the reduction of $\Omega _{\text {eff}}$ due to the factor $\mu ^{-1}$, within considered range of $\mu$. As expected, in both types of the dependence, growing confinement leads to the reduction of the dipole polarizability.

**Figure 6:**
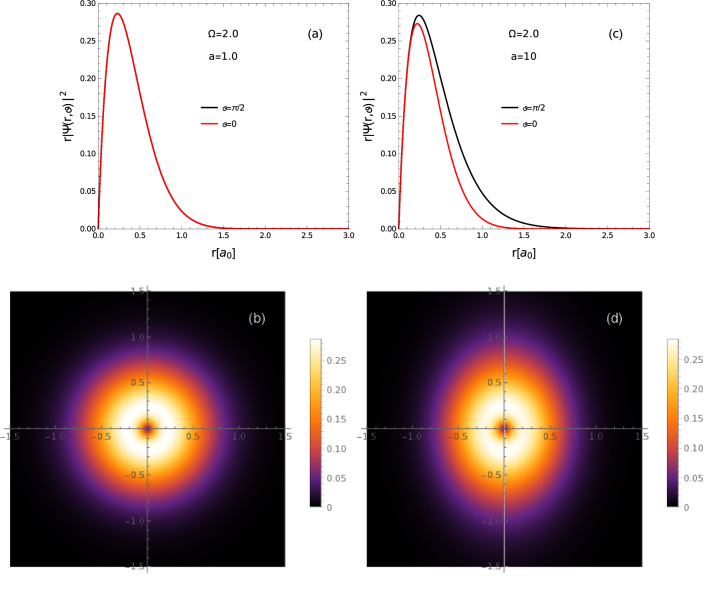
Ground state electron-hole density function (intracule density) of Mott-Wannier exciton for different levels of the deformation of confining potential: (**a**), (**b**) isotropic case ($a=1$); (**c**), (**d**) anisotropic case $a=10$.

In Fig. 6 is shown dependence on parameters of the confining potential of the electron-hole real-space density function (intracule density) defined as^42^ $|\Psi (\varvec{r})|^{2}=\int d\varvec{r}_ed\varvec{r}_h\delta \left[ \varvec{r}-(\varvec{r}_e-\varvec{r}_h)\right] |\Psi _{\text {exc}}(\varvec{r}_e,\varvec{r}_h)|^{2},$ where $\delta (\varvec{r})$ is the 2D Dirac delta function and $\Psi _{\text {exc}}$ is the excitonic wavefunction. The c.m. part of the wavefunction is taken for the ground state of the c.m. motion, that reduces after integration, the electron-hole density function to the relative motion density. The intracule density describes the probability density distribution for the relative vector $\varvec{r}_e-\varvec{r}_h$ of the electron-hole pair, as $\varvec{r}$. It can be interpreted as the probability of finding one of the particles (electron) in some region around the point $\varvec{r}$, when the position of second particle (hole) is fixed, that is given by $\rho (r,\vartheta )d\vartheta dr=|\Psi (r,\vartheta )|^{2}rd\vartheta dr$. The electron-hole pair densities corresponding to the ground state of the relative motion, for the case of isotropic confinement ($a=1$) are shown in Fig. 6a, b. We can see that density $\rho$ is also isotropic in this case. In Fig. 6c, d are given densities $\rho$ for highly anisotropic confinement ($a=10$). We can see that larger confinement in the *x* direction reduces the extension of $\rho$ in this direction and probabilities for distances between particles along $\vartheta \approx 0$ and $\vartheta \approx \pi /2$ directions differ, with preferred *y* direction. The extension in the *y* direction reduces the electron-hole Coulomb attraction, relatively to the *x* direction, that leads in turn to increase of the linear response of the exciton on external electric field, stretching the electron-hole pair more effectively in the *y* direction (due to weaker confinement), that is expressed in the difference in magnitudes of principal moments of the polarizability tensor.

**Figure 7:**
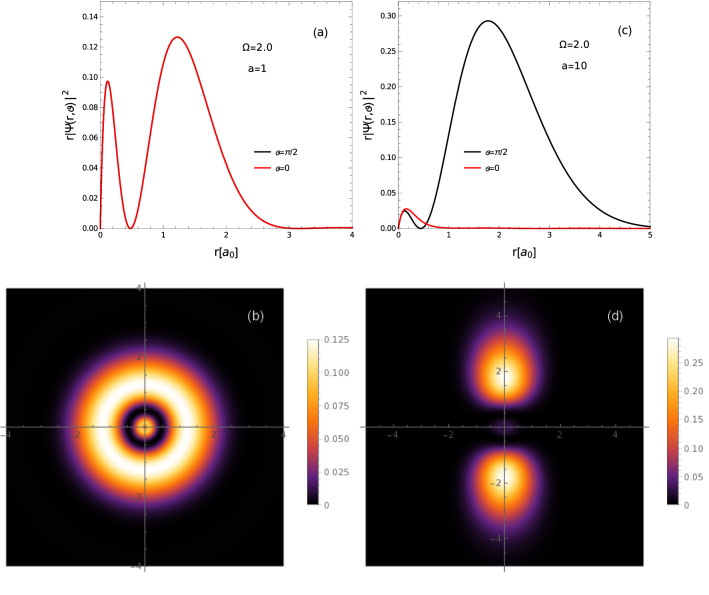
Electron-hole density function (intracule density) for first excited state of Mott-Wannier exciton for different levels of the deformation of confining potential: (**a**), (**b**) isotropic case ($a=1$); (**c**), (**d**) anisotropic case $a=10$.

Figure 7a, b present electron-hole densities corresponding to the first excited state of the relative motion of the electron-hole pair confined to the 2D Mott-Wannier exciton, for the case of isotropic external confinement ($a=1$). We can see that similarly to the ground state, the density $\rho$ is isotropic in this case. Figure 7c, d present densities $\rho$ for the highly anisotropic confinement ($a=10$). We can see that the anisotropy of the confinement is more visible in the electron-hole density function for excited states than for the ground state. This is a consequence of larger average extension of the system in excited state, leading to a stronger influence of the confining potential, that is an increasing function of the distance from the center of the dot. One can be also observed in Fig. 7d that due to the confinement, the two constituents of the exciton become well separated, which strongly reduces the probability of recombination.

### Summary

In the paper, we have presented an realistic example of the interplaying between stable external system and the quantum system subject to it. The exact theoretical study on the influence of the geometry of external quantum confinement on static dipole polarizability of Mott-Wannier exciton, formed close to the K-points of the first Brillouin zone of the TMDC monolayer material, has been presented. The properties of the quantum system expressed in terms of the static dipole polarizability as a linear response on the external electric field and the electron-hole density function have been linked with geometry of the external confinement. In particular, we have shown how both the magnitude and the anisotropy of the dipole polarizability may be effectively defined by the proper chose of the confinement strength and its geometry. One should be also noted that results obtained in this paper are universal in the sense that they are applicable for any MX$_2$ monolayer structure, after using appropriate scaling relations obtained in this paper, connecting the results calculated (or measured) for any given family member ML MX$_2$ with the results corresponding to arbitrary other ML TMDC structures with differing material parameters.

## Supplementary Information

Supplementary Information.

## Data Availability

The datasets used and/or analyzed during the current study are available from the corresponding author on reasonable request.
