# Supplementary material for: Geometry-modulated dipole polarizability of the two-dimensional Mott-Wannier excitons in gate-defined anisotropic quantum dot
Source: Sci Rep. 2022 Aug 30;12:14774. doi: 10.1038/s41598-022-19119-2 (PMC9427995; doi:10.1038/s41598-022-19119-2)
Supplement: Supplementary file 1 — Supplementary Information. [file 41598_2022_19119_MOESM1_ESM.pdf]

APPENDIX: ALGEBRAIZATION OF THE PROBLEM

After expressing the electron and the hole coordinates by the center-off mass $\boldsymbol{R} = [X, Y]$ and the relative motion $\boldsymbol{r} = [x, y]$ coordinates, according to

$$\boldsymbol{r}_e = \boldsymbol{R} + \frac{m_h}{M}\boldsymbol{r}, \quad \boldsymbol{r}_h = \boldsymbol{R} - \frac{m_e}{M}\boldsymbol{r}, \tag{A.1}$$

where $M = m_e + m_h$, one obtains from Eq. (10)

$$\mathsf{H}_{\text{exc}}(\boldsymbol{r}_e, \boldsymbol{r}_h) = \mathsf{H}_{\text{c.m.}}(\boldsymbol{R}(\boldsymbol{r}_e, \boldsymbol{r}_h)) + \mathsf{H}_{\text{rel}}(\boldsymbol{r}(\boldsymbol{r}_e, \boldsymbol{r}_h)), \tag{A.2}$$

where in atomic units,

$$\mathsf{H}_{\text{c.m.}}(\boldsymbol{R}) = E_g - \frac{1}{2M}\Big(\partial_X^2 + \partial_Y^2\Big) + \frac{1}{2}M\Big(\Omega_x^2 X^2 + \Omega_y^2 Y^2\Big) \tag{A.3}$$

and

$$\mathsf{H}_{\text{rel}}(\boldsymbol{r}) = -\frac{1}{2\mu}\Big(\partial_x^2 + \partial_y^2\Big) + \frac{1}{2}\mu\Big(\Omega_x^2 x^2 + \Omega_y^2 y^2\Big) + \boldsymbol{\eta}\cdot\boldsymbol{r} - \frac{1}{\varepsilon r}, \tag{A.4}$$

where $r = \sqrt{x^2 + y^2}$, $\boldsymbol{\eta} = \boldsymbol{F}/F_0$ and $\boldsymbol{F} = F[\cos\Phi, \sin\Phi]$ is the electric field vector. The c.m. Hamiltonian represents the sum of two independent linear oscillators. The solution to corresponding Schödinger equation is well known and may be found in any textbook for Quantum Mechanics. The relative motion Hamiltonian is transformed to the form

$$\mathsf{H} = -\nabla_r^2 - \frac{2Z}{r} + 2\tilde{\Omega}^2 r^2\Big(\frac{a^2}{1+a^2} + \frac{1-a^2}{1+a^2}\sin^2\vartheta\Big) + 2\tilde{\eta}r(\cos\Phi\cos\vartheta + \sin\Phi\sin\vartheta), \tag{A.5}$$

where $\mathsf{H} = 2\mu\mathsf{H}_{\text{rel}}, Z = \mu/\varepsilon, \tilde{\Omega} = \mu\Omega, \tilde{\eta} = \mu\eta, \Omega^2 = (\Omega_x^2 + \Omega_y^2)/2, a = \Omega_x/\Omega_y$. For further analysis we introduce following decomposition

$$\mathsf{H} = \mathsf{H}_0 + 2\frac{a^2}{1+a^2}\tilde{\Omega}^2 r^2 + 2\frac{1-a^2}{1+a^2}\tilde{\Omega}^2\mathsf{V}_a + 2\tilde{\eta}_x\mathsf{V}_x + 2\tilde{\eta}_y\mathsf{V}_y, \tag{A.6}$$

where $\mathsf{H}_0 = -\nabla_r^2 - 2Z/r, \mathsf{V}_x = r\cos\vartheta, \mathsf{V}_y = r\sin\vartheta, \mathsf{V}_a = r^2\sin^2\vartheta, \tilde{\eta}_x = \tilde{\eta}\cos\Phi, \tilde{\eta}_y = \tilde{\eta}\sin\Phi$. The diagonalization of H may be conveniently performed in the basis of the 2D discrete Coulomb Sturmians [33]. The basis is constructed from the functions

$$G_{nm}(r,\vartheta) = \Phi_m(\vartheta)\frac{S_{nm}^{(\xi)}(r)}{\sqrt{r}}, \quad \Phi_m(\vartheta) = \frac{e^{im\vartheta}}{\sqrt{2\pi}}, \tag{A.7}$$

where radial Coulomb Sturmians read

$$S_{nm}^{(\xi)}(r) = N_{nm}(r\xi)^{|m|+1/2}\mathrm{e}^{-r\xi/2}L_n^{(2|m|)}(r\xi), \tag{A.8}$$

where $L_n^{(\beta)}$ is generalized Laguerre polynomial $(n = 0, 1, 2, ...)$ and ξ is fixed parameter and

$$N_{nm} = \sqrt{\frac{n!}{(n + 2\mid m\mid)!}}. \tag{A.9}$$

The Sturmian functions form a complete basis well adjusted to the description of Coulomb-like problems. The Sturmian expansions in the case of Coulombic systems correctly reflect the behavior of the exact solutions and allow for a compact analytic-like expression of the wavefunction. Last but not least, relevant matrix elements may be expressed analytically. The 2D radial Sturmian functions satisfy the second-order differential equation

$$\left[-\frac{d^2}{dr^2} + \frac{m^2 - 1/4}{r^2}\right]S_{nm}^{(\xi)}(r) = \left[-\frac{\xi^2}{4} + \frac{(n + \mid m\mid + 1/2)\xi}{r}\right]S_{nm}^{(\xi)}(r) \tag{A.10}$$

and the orthogonality condition

$$\int_0^\infty S_{n'm}^{(\xi)}(r)\frac{1}{r}S_{nm}^{(\xi)}(r)dr = \delta_{n'n}. \tag{A.11}$$

Introducing the notation for matrix elements of any operator $\mathsf{P}(r,\vartheta)$,

$$\langle n'm'|\mathsf{P}|nm\rangle = \int_0^{2\pi} d\vartheta \int_0^{\infty} dr \Phi^*_{m'}(\vartheta) S^{(\xi)}_{n'm'}(r) \mathsf{P}(r,\vartheta) \Phi_m(\vartheta) S^{(\xi)}_{nm}(r) \tag{A.12}$$

and using following relations

$$\cos\vartheta\Phi_m = \frac{1}{2}(\Phi_{m-1}+\Phi_{m+1}), \;\; \sin\vartheta\Phi_m = \frac{i}{2}(\Phi_{m-1}-\Phi_{m+1}), \;\; \sin^2\vartheta\Phi_m = \frac{1}{4}(2\Phi_m - \Phi_{m-2} - \Phi_{m+2}) \tag{A.13}$$

one obtains

$$\langle n'm'|\mathsf{H}_0|nm\rangle = \delta_{m'm}\Big(\big[(n+\mid m\mid+\frac{1}{2}\xi) - 2Z\big]\delta_{n'n} - \frac{\xi^2}{4}\mathsf{B}^m_{n'n}\Big), \tag{A.14}$$

$$\langle n'm'|\mathsf{V}_x|nm\rangle = \frac{1}{2}\Big(\delta_{m',m-1}\mathsf{K}^m_{n'n} + \delta_{m',m+1}\mathsf{K}^{m+1}_{nn'}\Big), \tag{A.15}$$

$$\langle n'm'|\mathsf{V}_y|nm\rangle = \frac{i}{2}\Big(\delta_{m',m-1}\mathsf{K}^m_{n'n} - \delta_{m',m+1}\mathsf{K}^{m+1}_{nn'}\Big), \tag{A.16}$$

$$\langle n'm'|r^2|nm\rangle = \delta_{m'm}\mathsf{D}^m_{n'n}, \tag{A.17}$$

$$\langle n'm'|\mathsf{V}_a|nm\rangle = \frac{1}{2}\delta_{m'm}\mathsf{D}^m_{n'n} - \frac{1}{4}\Big(\delta_{m',m-2}\mathsf{T}^m_{n'n} + \delta_{m',m+2}\mathsf{T}^{m+2}_{nn'}\Big), \tag{A.18}$$

where the overlap matrix,

$$\mathsf{B}^m_{n'n} = \int_0^{\infty} dr S^{(\xi)}_{n'm}(r) S^{(\xi)}_{nm}(r) \tag{A.19}$$

and

$$\mathsf{K}^m_{n'n} = \int_0^{\infty} dr S^{(\xi)}_{n',m-1}(r)\, r\, S^{(\xi)}_{nm}(r), \tag{A.20}$$

$$\mathsf{D}^m_{n'n} = \int_0^{\infty} dr S^{(\xi)}_{n'm}(r)\, r^2 S^{(\xi)}_{nm}(r), \tag{A.21}$$

$$\mathsf{T}^m_{n'n} = \int_0^{\infty} dr S^{(\xi)}_{n',m-2}(r)\, r^2 S^{(\xi)}_{nm}(r). \tag{A.22}$$

Using properties of generalized Laguerre polynomials, all the integrals can be calculated analytically. They are given by

$$\mathsf{B}^m_{n'n} = \frac{N_{nm}}{N_{n'm}}\xi^{-1}\Big[-n'\delta_{n'-1,n} + (2n'+2\mid m\mid+1)\delta_{n'n} - (n'+2\mid m\mid+1)\delta_{n'+1,n}\Big], \tag{A.23}$$

$$\begin{aligned}\mathsf{K}^m_{n'n} = \frac{N_{n',m-1}}{N_{nm}}\xi^{-2}\Big[&-(n'+1)\delta_{n'+1,n} + 2\Big(2n'+\mid m\mid+\frac{1}{2}\Big)\delta_{n'n} - 6\Big(n'+\mid m\mid-\frac{1}{2}\Big)\delta_{n'-1,n} \\ &+2\Big(2n'+3\mid m\mid-\frac{5}{2}\Big)\delta_{n'-2,n} - (n'+2\mid m\mid-2)\delta_{n'-3,n}\Big],\end{aligned} \tag{A.24}$$

for $m \geqslant 1$, and $\mathsf{K}^{-|m|}_{n'n} = \mathsf{K}^{|m|+1}_{nn'}$, for $m \leqslant 0$.

$$\begin{aligned}\mathsf{D}^m_{n'n} = \frac{N_{nm}}{N_{n'm}}\xi^{-3}\Big[&-(n+1)(n+2)(n+3)\delta_{n',n+3} + 3(n+1)(n+2)(2n+2\mid m\mid+3)\delta_{n',n+2} \\ &-(n+1)\Big[n(n+2\mid m\mid) + 4(2n+2\mid m\mid+1)(n+\mid m\mid+1) + (n+2)(n+2\mid m\mid+2) \\ &+(n+1)(n+2\mid m\mid+1) + (2n+2\mid m\mid+3)^2\Big]\delta_{n',n+1} \\ &+\Big[4n(n+2\mid m\mid)(n+\mid m\mid) + (2n+2\mid m\mid+1)\big[(n+1)(6n+10\mid m\mid+1) \\ &+(2\mid m\mid-1)(n+2\mid m\mid-1)\big] + 4(n+1)(n+2\mid m\mid+1)(n+\mid m\mid+1)\Big]\delta_{n'n} \\ &-3(n+2\mid m\mid)\big[5n(n+2\mid m\mid)+4m^2+1\big]\delta_{n',n-1} + 3(n+2\mid m\mid)(n+2\mid m\mid-1)(2n+2\mid m\mid-1)\delta_{n',n-2} \\ &-(n+2\mid m\mid)(n+2\mid m\mid-1)(n+2\mid m\mid-2)\delta_{n',n-3}\Big],\end{aligned} \tag{A.25}$$

$$\begin{aligned}\mathsf{T}^m_{n'n} = \frac{N_{n',m-2}}{N_{nm}}\xi^{-3}\Big[&-(n'+1)\delta_{n'+1,n} + 2\Big(3n'+\mid m\mid+\frac{1}{2}\Big)\delta_{n'n} - 5(3n'+2\mid m\mid-2)\delta_{n'-1,n} \\ &+20\Big(n'+\mid m\mid-\frac{3}{2}\Big)\delta_{n'-2,n} - 5(3n'+4\mid m\mid-7)\delta_{n'-3,n} + 2\Big(3n'+5\mid m\mid-\frac{19}{2}\Big)\delta_{n'-4,n} \\ &-(n'+2\mid m\mid-4)\delta_{n'-5,n}\Big],\end{aligned} \tag{A.26}$$

for $m \geqslant 2$ and $\mathsf{T}^m_{n'n} = \mathsf{T}^{|m|+2}_{nn'}$, for $m \leqslant 0$ and $\mathsf{T}^1_{n'n} = \mathsf{D}^1_{n'n}$. Finally, the Hamiltonian matrix is given by

$$\begin{aligned}\langle n'm'|\mathsf{H}|nm\rangle = \delta_{m'm}\Big(&\big[(n+\mid m\mid+\frac{1}{2}\xi) - 2Z\big]\delta_{n'n} - \frac{\xi^2}{4}\mathsf{B}^m_{n'n} + \tilde{\Omega}^2\mathsf{D}^m_{n'n}\Big) \\ &+\tilde{\eta}\Big(\mathrm{e}^{i\Phi}\delta_{m',m-1}\mathsf{K}^m_{n'n} + \mathrm{e}^{-i\Phi}\delta_{m',m+1}\mathsf{K}^{m+1}_{nn'}\Big) \\ &+\frac{1}{2}\tilde{\Omega}^2\frac{a^2-1}{a^2+1}\Big(\delta_{m',m-2}\mathsf{T}^m_{n'n} + \delta_{m',m+2}\mathsf{T}^{m+2}_{nn'}\Big).\end{aligned} \tag{A.27}$$

The relative motion excitonic wavefunctions is given by

$$\Psi(r,\vartheta) = \sum_{m=-M'}^{M'}\sum_{n=0}^{N'} C_{nm}G_{nm}(r,\vartheta), \tag{A.28}$$

where M', N' are cut-of parameters. Energies E and the expansion coefficients $C_{nm} = \tilde{C}_{k(n,m)}$ are obtained from the generalized eigenvalue problem,

$$[\mathsf{H}]\{\tilde{C}\} = 2\mu E[\mathsf{B}]\{\tilde{C}\}, \tag{A.29}$$

where $[\mathsf{H}]$ and $[\mathsf{B}]$ is the Hamiltonian matrix and the overlap matrix, respectively. The matrix elements are ordered as $\langle k'|\mathsf{H}|k\rangle$, where for a given (n,m) the index $k(n,m) = (m+M')(N'+1)+n+1$, for $m = -M',...,M'$ and $n = 0,...,N'$. The matrix size, $L = (N'+1)(2M'+1)$. $\{\tilde{C}\}$ is the vector of coefficients $\tilde{C}_k$ $(k = 1,...,L)$. This ordering corresponds in fact to block structures ordered by number m. Introducing following matrices

$$\mathsf{A}^m_{n'n} = \big[(n+\mid m\mid+\frac{1}{2}\xi) - 2Z\big]\delta_{n'n} - \frac{\xi^2}{4}\mathsf{B}^m_{n'n} + \tilde{\Omega}^2\mathsf{D}^m_{n'n}, \tag{A.30}$$

$$\mathsf{P}^m_{n'n} = \tilde{\eta}\mathrm{e}^{i\Phi}\mathsf{K}^m_{n'n}, \quad \mathsf{W}^m_{n'n} = \frac{1}{2}\tilde{\Omega}^2\frac{a^2-1}{a^2+1}\mathsf{T}^m_{n'n}, \tag{A.31}$$

we can write matrices $[\mathsf{H}]$ and $[\mathsf{B}]$ explicitly in the block forms as

$$[\mathsf{H}]_{m'm} = \delta_{m',m+2}\tilde{\mathsf{W}}^m + \delta_{m',m+1}\tilde{\mathsf{P}}^m + \delta_{m'm}\mathsf{A}^m + \delta_{m',m+1}\mathsf{P}^{m+1} + \delta_{m',m+2}\mathsf{W}^{m+2}, \tag{A.32}$$

$$[\mathsf{B}]_{m'm} = \delta_{m'm}\mathsf{B}^m, \tag{A.33}$$

where corresponding blocks have a dimension $(N'+1)\times(N'+1)$, the tilde means a hermitian conjugate of the matrix and $(m', m = -M', ..., M')$. Corresponding vector of coefficients may be written as $\{\tilde{C}\} = [\mathsf{J}^{(-M')}, ..., \mathsf{J}^{(M')}]^T$, where every *block* component $\mathsf{J}^{(m)}$ contains $N'+1$ coefficients. The m-th equation of the linear system (A.29) reads

$$\tilde{\mathsf{W}}^m\mathsf{J}^{(m-2)} + \tilde{\mathsf{P}}^m\mathsf{J}^{(m-1)} + \mathsf{A}^m\mathsf{J}^{(m)} + \mathsf{P}^{m+1}\mathsf{J}^{(m+1)} + \mathsf{W}^{m+2}\mathsf{J}^{(m+2)} = 2\mu E\mathsf{B}^m\mathsf{J}^{(m)}. \tag{A.34}$$

At the first step we find the eigenvalue decompositions of the blocks B^m,

$$\mathsf{B}^m = \Gamma_m\mathsf{B}^m_D\Gamma^\dagger_m, \quad \mathsf{B}^m_D = \mathsf{B}^{1/2}_m\mathsf{B}^{1/2}_m, \tag{A.35}$$

where the diagonal matrix B^m_D and the transformation matrix Γ_m are obtained directly by diagonalization of B^m. The last decomposition of B^m_D as a square of a real matrix $\mathsf{B}^{1/2}_m$ is allowed since the overlap matrix is positively defined. We note that matrix Γ_m is unitary since B^m is a hermitian matrix. Finally we can write,

$$\mathsf{B}^m = \Gamma_m\mathsf{B}^{1/2}_m\mathsf{B}^{1/2}_m\Gamma^\dagger_m. \tag{A.36}$$

Substituting the matrix B^m given by the last equation in the r.h.s. of Eq. (A.34) and myltiplying the equation from the left by $\Gamma^\dagger_m$ and next by $(\mathsf{B}^{1/2}_m)^{-1} \equiv \mathsf{B}^{-1/2}_m$ and introducing new coefficients

$$\mathsf{J}^{(m)}_t = \mathsf{B}^{1/2}_m\Gamma^\dagger_m\mathsf{J}^{(m)} \tag{A.37}$$

and transformed matrices

$$\mathsf{A}^m_t = \mathsf{B}^{-1/2}_m\Gamma^\dagger_m\mathsf{A}^m\Gamma_m\mathsf{B}^{-1/2}_m, \quad \mathsf{P}^m_t = \mathsf{B}^{-1/2}_{m-1}\Gamma^\dagger_{m-1}\mathsf{P}^m\Gamma_m\mathsf{B}^{-1/2}_m, \quad \mathsf{W}^m_t = \mathsf{B}^{-1/2}_{m-2}\Gamma^\dagger_{m-2}\mathsf{W}^m\Gamma_m\mathsf{B}^{-1/2}_m, \tag{A.38}$$

we obtain the equation

$$\tilde{\mathsf{W}}^m_t\mathsf{J}^{(m-2)}_t + \tilde{\mathsf{P}}^m_t\mathsf{J}^{(m-1)}_t + \mathsf{A}^m_t\mathsf{J}^{(m)}_t + \mathsf{P}^{m+1}_t\mathsf{J}^{(m+1)}_t + \mathsf{W}^{m+2}_t\mathsf{J}^{(m+2)}_t = 2\mu E\mathsf{J}^{(m)}_t, \tag{A.39}$$

with the unit matrix on the r.h.s. In this manner the original generalized eigenvalue problem (A.29) is transformed to a simple eigenvalue problem with hermitian 5-diagonal block matrix,

$$[\mathsf{H}_t]\{\tilde{C}_t\} = 2\mu E\{\tilde{C}_t\}, \tag{A.40}$$

$$[\mathsf{H}_t]_{m'm} = \delta_{m',m+2}\tilde{\mathsf{W}}^m_t + \delta_{m',m+1}\tilde{\mathsf{P}}^m_t + \delta_{m'm}\mathsf{A}^m_t + \delta_{m',m+1}\mathsf{P}^{m+1}_t + \delta_{m',m+2}\mathsf{W}^{m+2}_t, \tag{A.41}$$

where $\{\tilde{C}_t\} = [\mathsf{J}^{(-M')}_t, ..., \mathsf{J}^{(M')}_t]^T$. At the next step the matrix $[\mathsf{H}_t]$ is transformed to the 3-diagonal (scalar) form using the Lanczos algorithm and finally diagonalized using standard numerical procedures. The original coefficients are given by

$$\mathsf{J}^{(m)} = \Gamma_m\mathsf{B}^{-1/2}_m\mathsf{J}^{(m)}_t. \tag{A.42}$$
